# Vitamin C deficiency improves somatic embryo development through distinct gene regulatory networks in *Arabidopsis*

**DOI:** 10.1093/jxb/eru330

**Published:** 2014-08-23

**Authors:** Michael G. Becker, Ainsley Chan, Xingyu Mao, Ian J. Girard, Samantha Lee, Mohamed Elhiti, Claudio Stasolla, Mark F. Belmonte

**Affiliations:** ^1^Department of Biological Sciences, University of Manitoba, Winnipeg, MB, R3T2N2, Canada; ^2^Department of Botany, Faculty of Science, Tanta University, Tanta, 31527, Egypt; ^3^Department of Plant Science, University of Manitoba, Winnipeg, MB, R3T2N2, Canada

**Keywords:** *Arabidopsis thaliana*, ascorbic acid, gene regulatory networks, redox, somatic embryogenesis, transcriptome

## Abstract

Changes in the endogenous ascorbate redox status through genetic manipulation of cellular ascorbate levels were shown to accelerate cell proliferation during the induction phase and improve maturation of somatic embryos in *Arabidopsis*. Mutants defective in ascorbate biosynthesis such as *vtc2-5* contained ~70 % less cellular ascorbate compared with their wild-type (WT; Columbia-0) counterparts. Depletion of cellular ascorbate accelerated cell division processes and cellular reorganization and improved the number and quality of mature somatic embryos grown in culture by 6-fold compared with WT tissues. To gain insight into the molecular mechanisms underlying somatic embryogenesis (SE), we profiled dynamic changes in the transcriptome and analysed dominant patterns of gene activity in the WT and *vtc2-5* lines across the somatic embryo culturing process. Our results provide insight into the gene regulatory networks controlling SE in *Arabidopsis* based on the association of transcription factors with DNA sequence motifs enriched in biological processes of large co-expressed gene sets. These data provide the first detailed account of temporal changes in the somatic embryo transcriptome starting with the zygotic embryo, through tissue dedifferentiation, and ending with the mature somatic embryo, and impart insight into possible mechanisms for the improved culture of somatic embryos in the *vtc2-5* mutant line.

## Summary

Depletion of cellular vitamin C improves somatic embryogenesis in *Arabidopsis*. Improved embryo number and quality is through changes in gene regulatory network activation and cellular architecture.

## Introduction

Somatic embryogenesis (SE) in higher plants is one of the best examples of cellular totipotency and is commonly used as a model system to study the complex events that underlie plant embryogenesis (Kurczyńska *et al.*, 2007). Briefly, the process involves somatic cells generating mature embryos *in vitro*. This can occur directly from somatic cells or indirectly through an intermediate callus phase. Once the new somatic embryos begin forming, their development is reminiscent of that of zygotic embryogenesis (ZE). Many excellent reviews describing SE already exist (Zimmerman 1993; von Arnold *et al.*, 2002; Harada *et al.*, 2010).

**Fig. 1.**
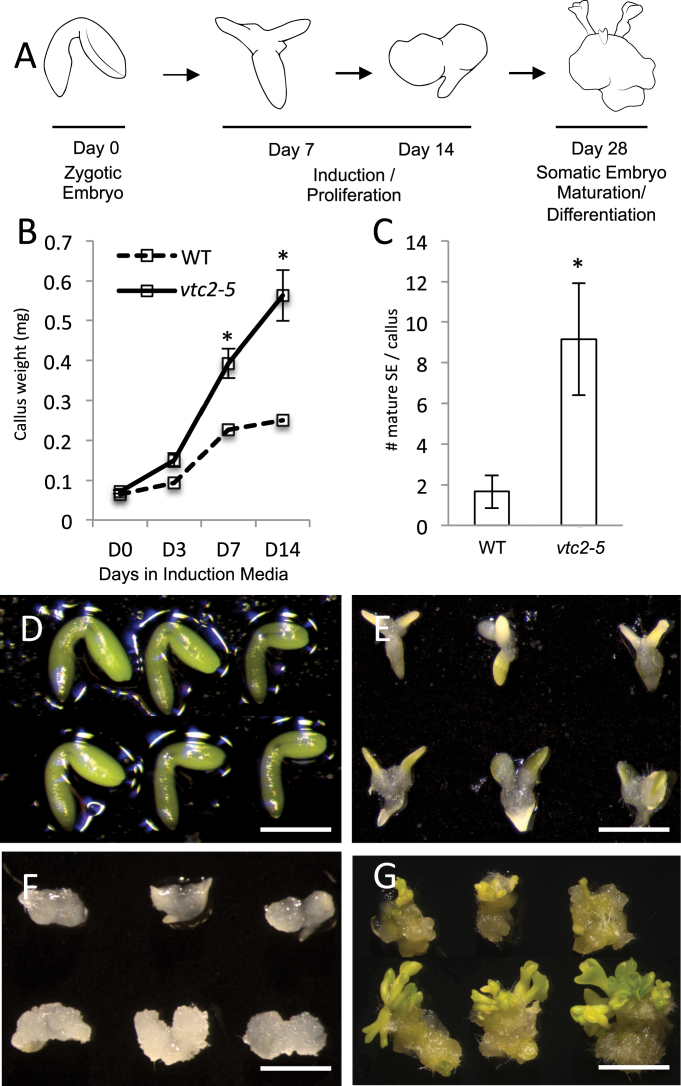
*VTC2* depletion improves SE in *Arabidopsis*. (A) Schematic representation of the SE process in *Arabidopsis*. (B) Callus size significantly increases in *vtc2-5* (solid line) compared with the WT [dashed line; means±standard deviation (SD)]. (C) Number of mature somatic embryos formed per callus (means±SD). Asterisks above the bar indicate significant difference from control values (*P*<0.05) at the same sampling time. (D) Zygotic embryos in WT (top) and *vtc2-5* (bottom). Bar, 1mm. (E) Representative embryos at 7 d on induction medium. Bar, 1.5mm. (F) At 14 d on induction medium, proliferation of the embryo is much more profound in *vtc2* compared with WT. Bar, 3.5mm. (G) Embryos were transferred to a medium lacking 2,4-D. Here, we show three representative clusters of somatic embryos at 14 d of maturation. Bar, 5mm. (This figure is available in colour at *JXB* online.)

In *Arabidopsis thaliana*, indirect SE can be divided into two distinct stages: induction and maturation (Fig. 1). Bent-cotyledon-stage zygotic embryos are cultured on a medium enriched with the growth-stimulating phytohormone auxin [2,4-dichlorophenoxyacetic acid (2,4-D)] for 14 d. Cells responsive to the induction medium begin to proliferate and dedifferentiate into an unorganized callus tissue. This begins in the cotyledons of the zygotic embryos, before spreading along the entire embryo axis (Raghavan, 2004). The callus then generates young embryos before being transferred to medium without 2,4-D to stimulate embryo maturation (Bassuner *et al.*, 2007). While SE is a viable means to produce large numbers of embryos from a single explant, the embryos that are produced can sometimes exhibit low quality and regeneration capabilities (Bassuner *et al.*, 2007; Kurczyńska *et al.*, 2007; Elhiti *et al.*, 2010). Since SE is used as a quick and efficient tool in the production of genetically identical plants with desirable traits for agricultural and commercial applications, new methods to improve somatic embryo quality and production are needed.

Previous successful attempts to improve somatic embryo quality and production have focused on culture environment optimization using antioxidants or antioxidant inhibitors (Belmonte and Yeung, 2004; Belmonte and Stasolla, 2007; Stasolla *et al.*, 2008). However, these strategies relied on exogenous supplementation to the culture medium and required extensive trials to optimize growth conditions. Thus, identifying genetic means to improve somatic embryo development through changes in cellular status should reduce error and achieve large numbers of quality somatic embryos.

Alterations in cellular redox status have been shown to play a role in embryo development (Cairns *et al.*, 2006), and manipulations to redox state *in vivo* and *in vitro* can alter somatic embryo growth rates and regeneration frequencies (Bassuner *et al.*, 2007; Elhiti *et al.*, 2010). It is thought that embryogenesis relies on a series of redox switches that transform the cellular environment from a more reduced to a more oxidized state. These changes in antioxidant status are accompanied by large changes in gene activity during tissue patterning, differentiation, and the accumulation of storage reserves. In general, a more reduced state is associated with increased cellular proliferation, while a more oxidized redox pool enhances embryo maturation. It has been demonstrated previously that modifications in the cellular redox state are responsible for embryo development *in vitro* and *in vivo* (Belmonte *et al.*, 2005; Stasolla *et al.*, 2008).

**Fig. 2.**
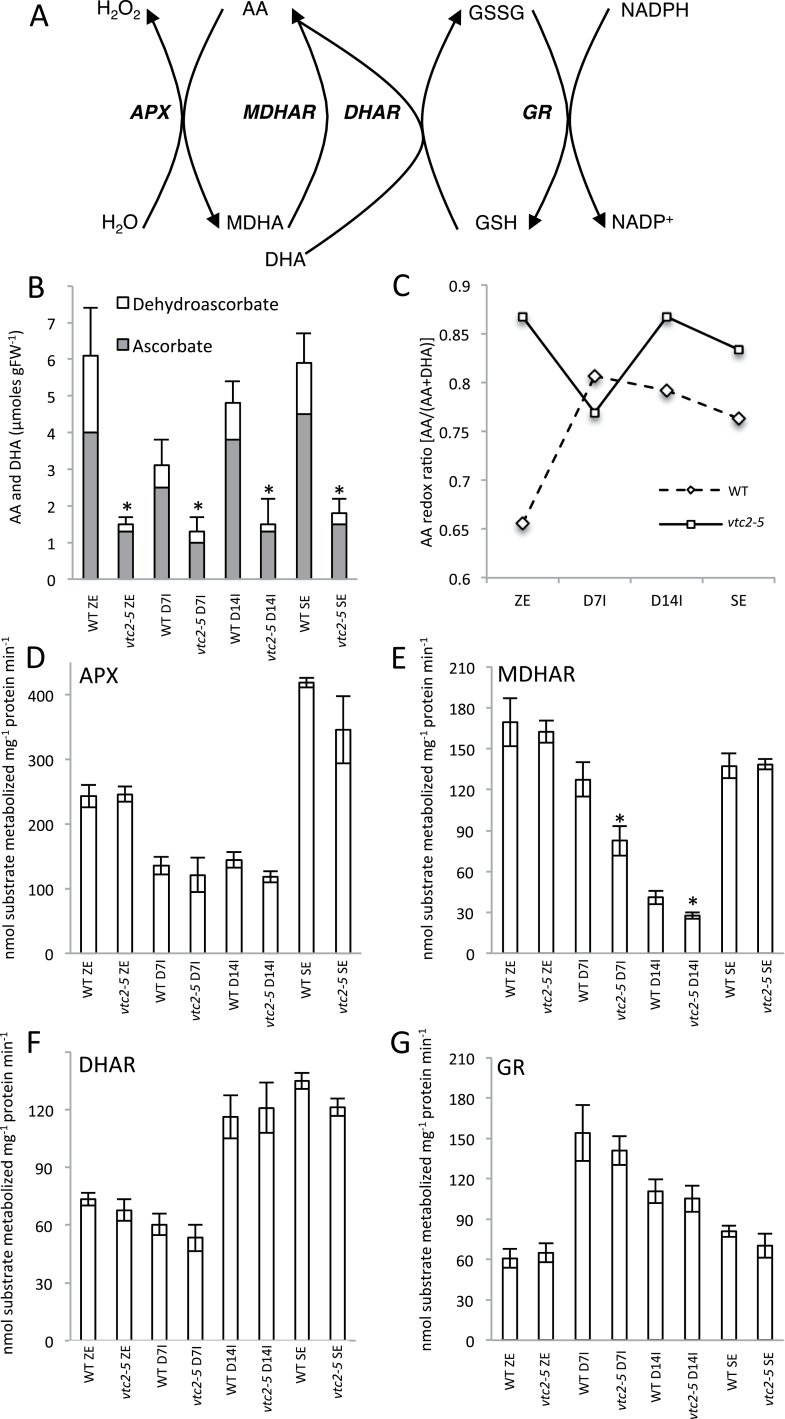
Effect of AA deficiency on cellular redox status during SE in WT Col-0 and *vtc2-5*. (A) Schematic diagram of the ascorbate–glutathione cycle. APX, ascorbate peroxidase; ASC, ascorbic acid; DHA, dehydroascobate; MDHA, monodehydroascorbate; MDHAR, monodehydroascorbate reductase; DHAR, dehydroascorbate reductase; GSH, glutathione; GSSG, glutathione disulfide; GR, glutathione reductase. (B) Endogenous cellular levels of AA and DHA during somatic embryogenesis. (C) AA redox ratio throughout somatic embryogenesis. (D) Cellular levels of APX. (E) MDHAR levels. (F) Cellular levels of DHAR. (G) Endogenous levels of GR during somatic embryogenesis. Asterisks above bars indicate a significant difference from WT control values (*P*<0.05) at the same sampling time.

Reactive oxygen species are natural by-products of cellular metabolism but can be harmful to the cell if not detoxified. The removal of reactive oxygen species is accomplished largely by the ascorbate–glutathione cycle (Fig. 2A), in which vitamin C [ascorbic acid (AA)] plays an integral role. AA exists in two forms: reduced ascorbate (ASC) and oxidized dehydroascorbate (DHA). When the redox pool of developing somatic embryos was disrupted by the application of the glutathione biosynthesis inhibitor buthionine sulfoximine, increased regeneration frequencies were observed (Stasolla *et al.*, 2008). This coincided with a decrease in the ratio of ASC/DHA and a decline in the total AA pool (Stasolla *et al.*, 2008). However, while inhibitors can be useful in manipulating redox state, and therefore the progress of SE, they can cause additional pleiotropic effects that can confound results.

This current study focused on AA depletion and its effect on SE. A number of AA-deficient mutants have been identified through genetic screens (Conklin *et al.*, 1996; Jander *et al.*, 2002), and have contributed to our understanding of AA biosynthesis. Four AA-deficient mutants—*vitamin C deficient 1* (*vtc1*), *vtc2*, *vtc3*, and *vtc4*—have been shown to affect a number of developmental programmes, including flowering time (Kotchoni *et al.*, 2009), root architecture (Olmos *et al.*, 2006), and cellular antioxidant status (Colville and Smirnoff, 2008). *VTC2* and its homologue, *VTC5*, encode the enzyme GDP-l-galactose phosphorylase, which is responsible for the first committed step to ascorbate synthesis in the l-galactose pathway, the primary route of AA biosynthesis in plants (Linster and Clarke, 2008). Unless supplemented with vitamin C, *vtc2 vtc5* double mutants do not survive (Dowdle *et al.*, 2007; Linster and Clarke 2008) and no viable mutant completely deficient in vitamin C has ever been discovered (De Tullio and Arrigoni, 2004). This suggests that AA is essential to plant growth and development.

Our results showed an increase in proliferation rates during SE induction and increased regeneration frequencies in the AA-deficient mutant *vtc2-5*. Changes in expression profiles controlling complex biological processes such as abiotic stress response, lipid metabolism, sugar signalling, and photosynthesis were observed from microarray analyses at each developmental stage of SE. New predictive transcriptional modules regulating major cellular processes active during the development and production of somatic embryos are also proposed.

## Materials and methods

### Plant material and growth

Seeds were surface sterilized and incubated for 3 d at 4 °C on half-strength Murashige and Skoog medium, pH 5.7, solidified with 1% Phytagel under sterile conditions. Plants were transferred to soil (Sunshine Mix #1) and grown at 22 °C under long-day conditions (16h light).

### Identification and characterization of plants with a T-DNA insert in VTC2

Wild-type (WT) *A. thaliana* [ecotype Columbia-0 (Col-0)] and T-DNA insertion lines were obtained from the Arabidopsis Biological Resource Center. Two independent SALK lines, *vtc2-5* (SALK_146824) and *vtc2-6* (SALK_076245), were screened with forward and reverse gene-specific primers and a left border insert-specific primer. Each insertion mutant line was verified as homozygous using PCR with primers specific for each T-DNA insertion. These primer sequences are listed in Supplementary Table S1 at *JXB* online. The positions of T-DNA insertion sites are shown in Fig. S1A at *JXB* online. Homozygous mutants (*vtc2-5* and *vtc2-6)* were identified by PCR and confirmed as having reduced levels of *VTC2* mRNA compared with Col-0 using allele-specific primers and quantitative reverse transcription-PCR (qRT-PCR) (Figs S1B and S2B at *JXB* online.).

### Culture treatments

*Arabidopsis* SE was carried out as reported by Bassuner *et al.* (2007). Briefly, zygotic embryos at the bent-cotyledon stage of development were dissected by hand and plated on an induction medium containing 2,4-D (Sigma-Aldrich) for 14 d to induce callus formation. Explants were then transferred to hormone-free maturation medium for 14 d and scored for the number of embryos that had formed (as indicated by the presence of a root and shoot apex). For 2,4-D treatments, 0, 1.2, 2.5, and 4.5 µM 2,4-D (filter sterilized) was supplemented in the induction medium. To rescue the *vtc2* phenotype, the induction medium was supplemented with filter-sterilized 0, 10, 100, or 1000 µM ascorbate (Sigma-Aldrich). At least 50 embryos were cultured per treatment in three replicates and repeated three to five times. Tukey’s post-hoc test for multiple variance was used to compare differences between treatments and control.

### Histological analyses, localization studies, and ASC metabolism

Structural studies using light and electron microscopy during SE were conducted as reported by Yeung (1999) and Elhiti *et al.* (2013), respectively. Localization of SHOOTMERISTEMLESS (STM) was performed by RNA *in situ* hybridization using the procedure outlined by Belmonte *et al.* (2006). Measurements of endogenous ASC and the enzyme activity of ascorbate peroxidase (APX), dehydroascorbate reductase (DHAR), glutathione reductase (GR), and monodehydroascorbate reductase (MDHAR) were carried out according to the methods of Belmonte *et al.* (2006) and Stasolla and Yeung (2007).

### Microarray and bioinformatics analyses

RNA was extracted from bent-cotyledon zygotic embryos, and somatic embryos at 7 d of induction (D7I), 14 d of induction (D14I) and 14 d of maturation (mature somatic embryos with a distinct embryonic axis) using Ambion® Plant RNA Reagent (Life Technologies, Carlsbad, California, USA). DNA contamination was removed with an Ambion® Turbo DNA-free™ kit (Life Technologies, Carlsbad, CA, USA) according to the manufacturer’s instructions. RNA concentration was measured with a Nanodrop 2000c spectrophotometer and RNA quality was assessed using an Agilent RNA 6000 Pico Chip (Agilent Technologies; Santa Clara, CA, USA) on an Agilent 2100 Bioanalyzer. Amplification and labelling were performed using 3 µg of input RNA into the Enzo® Single-Round RNA Amplification and Biotin Labeling System (Life Technologies, Farmingdale, NY, USA). The quality of amplified biotin-labelled antisense RNA was also assessed using an Agilent RNA 6000 Pico Chip on the Agilent 2100 Bioanalyzer. Two biological replicates were conducted for each genotype and used for microarray gene expression analysis according to the methods of Brady *et al.* (2007), Le *et al.* (2010), and Belmonte *et al.* (2013) with some modifications. Amplified, labelled, and fragmented RNA was then hybridized to the Affymetrix ATH1 Arabidopsis Genome Array following the manufacturer’s instructions, and as described by Le *et al.* (2010). Raw images (CEL format) were generated with Affymetrix GeneChip Operating Software (GCOS).

The number of mRNAs present in each tissue was determined using MAS 5.0 Software (Affymetrix, Santa Clara, CA, USA). An mRNA was considered present (‘P’) only if it was called ‘P’ in both biological replicates. For global comparisons of gene activity and quantitative analyses of gene expression, GeneChip data were normalized using the Robust Multichip Average (RMA) method (Dai *et al.*, 2005). Correlation between RMA-normalized biological replicates was then tested and shown to have an average correlation of 0.97 (with a range from 0.95 to 0.99) for WT tissues while all biological replicates of *vtc2-5* were 0.99 based on a Pearson’s correlation (Supplementary Dataset S1 at *JXB* online). RMA-normalized data was then clustered hierarchically using the pvclust package with default settings in R (http://cran.r-project.org/web/packages/pvclust/pvclust.pdf). To determine if genes were differentially expressed, a multiway significance analysis of microarrays test was carried out (Tusher *et al.*, 2001) and scores were analysed in terms of false discovery rate (FDR; *q* values). Only gene probes having an adjusted *P* value (FDR; Benjamini and Hochberg, 1995) of <0.05 and an absolute difference in expression fold change of 2 or greater were selected. A list of mRNAs that showed a 2-fold or higher change between WT and *vtc2-5* tissues at one stage of development are found in Supplementary Dataset S1; over-representation for gene ontology (GO) terms are in Supplementary Dataset S2 at *JXB* online and DNA sequence motifs are found in Supplementary Dataset S3 at *JXB* online.

Dominant patterns (DPs) of expression were identified according to the methods of Brady *et al.* (2007) and Belmonte *et al.* (2013) with the following modifications. Averaged RMA-normalized data from each tissue and genotype were filtered to remove probe sets with an RMA value ≤30. The filtered data were clustered using the fuzzy K means (FKM) implementation FANNY (http://cran.r-project.org/web/packages/cluster/cluster.pdf) with a K value of 15 and a cluster membership (*m* value) of 0.44. If clusters showed significant similarity (Pearson’s correlation coefficient), they were grouped together, resulting in 13 different RNA accumulation patterns. The mRNAs with a Pearson’s correlation of 0.85 or above were then assigned to each cluster with an average of 1146 mRNAs and a range from 138 to 2443. Lists of mRNAs assigned to each DP are found in Supplementary Dataset S1, while GO terms enrichment and genes belonging to each GO terms are found in Supplementary Dataset S2; DNA sequence motifs are provided in Supplementary Dataset S3. Putative transcriptional modules were then identified as described by Belmonte *et al.* (2013). Finally, to generate the GO term enrichment heatmaps, the data were log_10_ transformed and imported into TMeV (Saeed *et al.*, 2006).

### qRT-PCR experiments

qRT-PCR was used to validate and confirm selected genes of microarray data and to measure relative mRNA levels. The relative abundance of mRNA was analysed with the 2^–∆∆CT^ method (Livak and Schmittgen, 2001). Primers are listed in Supplementary Table S1.

### Microarray data accession numbers

All of the microarray data generated in this study can be found in the Gene Expression Omnibus (http://www.ncbi.nlm.nih.gov/geo/) under the series GSE48915 containing data from 16 individual samples.

## Results

### Response of vtc2-5 and vtc2-6 to SE

SE capability in *vtc2-5* and *vtc2-6* was compared with their WT counterpart (Fig. S1C–E). The number of immature zygotic embryos capable of producing callus tissue was almost 100% in *vtc2-5* and 85% in *vtc2-6* compared with an average of 65% in the WT (Fig. S1C). Callus tissue also appeared to develop faster in the AA-deficient mutant and increased in size on a g per fresh weight (FW) basis by almost 3-fold by the completion of induction (Fig. 1B). The number of calli capable of producing embryos increased by 15% in *vtc2-6* and by 35% in *vtc2-5* compared with the WT (Fig. S1D). The number of embryos produced by each responsive explant was greater in tissues defective in *VTC2* transcript levels (Fig. 1C), with over 30% of the explants generating more than five embryos compared with almost 5% in WT explants (Fig. S1E). These results suggested that *vtc2* mutants are able to generate calli faster, and these calli in turn are more embryogenic competent. Thus, attention was focused on *vtc2-5* to gain further insight into the role of AA in SE. Morphological differences between WT and *vtc2-5* cultures during the SE process are shown in Fig. 1D–G.

To reverse the effect of the mutant phenotype, the induction medium was supplemented with various concentrations of ascorbate (Fig. S2A). When ascorbate was supplied to the induction medium in the presence of 2,4-D, there was no appreciable difference in the number of zygotic embryos forming callus in the WT or *vtc2-5* line. When *vtc2* tissues were grown in the presence of ASC, their somatic embryo-forming capacity was reduced, a phenotypic effect that more closely resembled that of WT tissues.

To examine whether exogenous applications of 2,4-D affected the induction process, the induction medium was supplemented with various concentrations of this phytohormone (Fig. S3 at *JXB* online). Interestingly, a very small number of embryos from *vtc2* plants were able to produce callus in the absence of 2,4-D but were unable to produce somatic embryos (Fig. S3A, B). Figure S3C shows that the number of somatic embryos produced was dependent on the concentration of 2,4-D and was always more pronounced in tissues deficient in AA compared with WT tissues.

### Effect of *vtc2* on endogenous AA levels and changes in cellular AA redox homeostasis

A schematic diagram of the ascorbate–glutathione redox cycle is shown in Fig. 2A. To further validate *vtc2-5* as being deficient in cellular AA, we quantified endogenous ASC and DHA levels during the SE process (Fig. 2B). Total and reduced ASC levels were significantly lower (average ~70%) in *vtc2* compared with the WT tissues, supporting previous reports of *vtc2* mutants that also show an ~70% decrease in endogenous AA levels (Conklin *et al.*, 2000). There was a marked decrease in total AA levels in WT D7I tissues before gradually increasing to near-zygotic embryo levels by the mature somatic embryo stage (Fig. 2B), whereas the total AA levels remained low throughout the SE process in *vtc2* tissues. The ASC redox ratio also showed differences between the two genotypes (Fig. 2C). Notably, the redox ratio in *vtc2* zygotic embryos was more reduced compared with WT zygotic embryos.

Next, the activity of several redox enzymes, including APX, MDHAR, DHAR, and GR in developing control and *vtc2* mutant tissues were quantified (Fig. 2D–G). There were no significant differences in antioxidant enzyme activity between WT and *vtc2* tissues except for MDHAR during somatic embryo induction (Fig. 2E). However, differences in enzyme activity were prevalent across the SE process in either WT or *vtc2* when compared with the same genotype. For example, APX and DHAR activity was prevalent at the bent-cotyledon and mature somatic embryo stage, while the activity of GR increased profoundly in D7I somatic embryos. While changes in endogenous AA levels were recorded in *vtc2-5* compared with WT tissues, changes in the cellular redox ratio were probably not a result of redox cycling enzymes.

### Structural features of WT and *vtc2* tissues

**Fig. 3.**
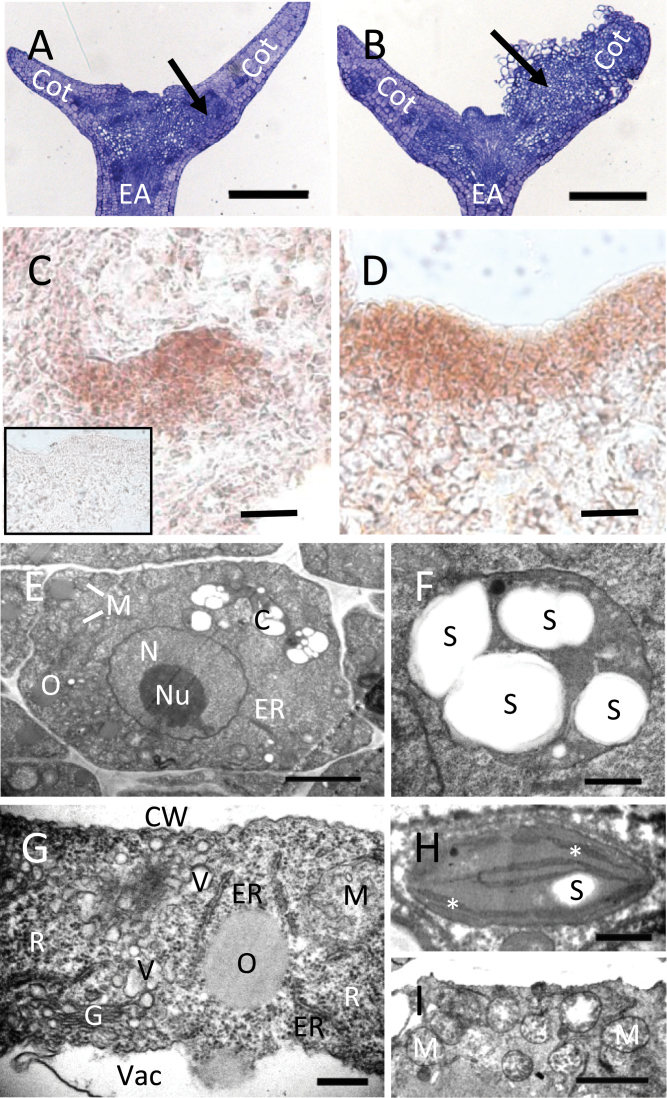
Structural features of WT and *vtc2* tissues during SE. (A, B) Medial longitudinal sections of a explants at D7I (+2,4-D) showing cell division within the cotyledon. EA, embryo axis; Cot, cotyledon. Arrows show areas of callus initiation. Bars, 250 µm. (A) WT embryo with evidence of some cell division in the cotyledon. (B) In *vtc2-5*, tissues were re-organizing and beginning to develop callus in the cotyledon. (C, D) *In situ* hybridization of *SHOOTMERISTEMLESS* in proliferating tissue at D7I in WT (C) and *vtc2-5* (D) using an anti-*STM* probe (sense probe, inset). Bars, 20 µm. (E–I) TEM images of WT versus *vtc2* somatic embryos during induction. (E) A typical whole cell from a WT somatic embryo. Note the lack of mitochondria and endomembrane system relative to *vtc2*. N, nucleus; Nu, nucleolus; M, mitochondria; O, oil body; C, chloroplast; ER, endoplasmic reticulum. Bar, 2 µm. (F) A chloroplast from a WT SE showing multiple starch granules (S). Bar, 500nm. (G) Extensive endomembranous structures and ribosomes (R) are found in the cytoplasm of a *vtc2* somatic embryo cell. G, Golgi; V, vesicle; ER, endoplasmic reticulum; M, mitochondria; O, oil body; CW, cell wall; V, vacuole. Bar, 300nm. (H) A chloroplast from a *vtc2* somatic embryo. Starch (S) is rare and smaller than in the WT. Thylakoid membranes are indicated by an asterisk. Bar, 500nm. (I) Many mitochondria (M) in the cytoplasm of a *vtc2* somatic embryo cell. Bar, 2 µm. (This figure is available in colour at *JXB* online.)

To determine if AA deficiency produces an effect on the morphology of somatic embryos, structural studies were performed. No appreciable morphological differences were observed in the harvested bent-cotyledon zygotic embryos (Fig. 1D), but notable changes in morphology and histology were apparent by D7I in the presence of auxin (Figs 1E and 3A, B). Figure 3B shows many cells undergoing cell division in the cotyledons compared with the WT (Fig. 3A), which appears to have fewer actively dividing cells. Since *vtc2* tissues appeared to proliferate faster compared with WT (Fig. 1B), the expression domain of *STM* in proliferating tissues at D7I at the edge of the cotyledon was examined using a marker used previously to detect improved somatic embryo capacity *in vitro* (Elhiti *et al.*, 2010). The expression domain of *STM* in D7I tissues was restricted to only a few cell layers (Fig. 3C) compared with *vtc2*, which had a larger and less-restricted STM domain when tissues were subjected to the induction medium towards the edges of the explants (Fig. 3D).

A cortical WT cell of a somatic embryo had all the basic cellular features (Fig. 3E), including a nucleus, mitochondria, endomembranous structures, and chloroplasts with starch grains (Fig. 3F). Extensive endomembranous and ribosomal activity in *vtc2* cortical cells (Fig. 3G) suggested intracellular remodelling that might be associated with dedifferentiation. While chloroplasts were present in *vtc2* tissues, they usually contained little to no starch (Fig. 3H), and the cytoplasm was also characterized by many more mitochondria (Fig. 3I) than were found in the WT. Anatomical evidence suggested that cells were de-differentiating and actively dividing during SE, and that this was happening earlier and to a greater extent in the *vtc2* mutant.

### Transcriptome analysis of somatic embryo development in WT tissues

**Fig. 4.**
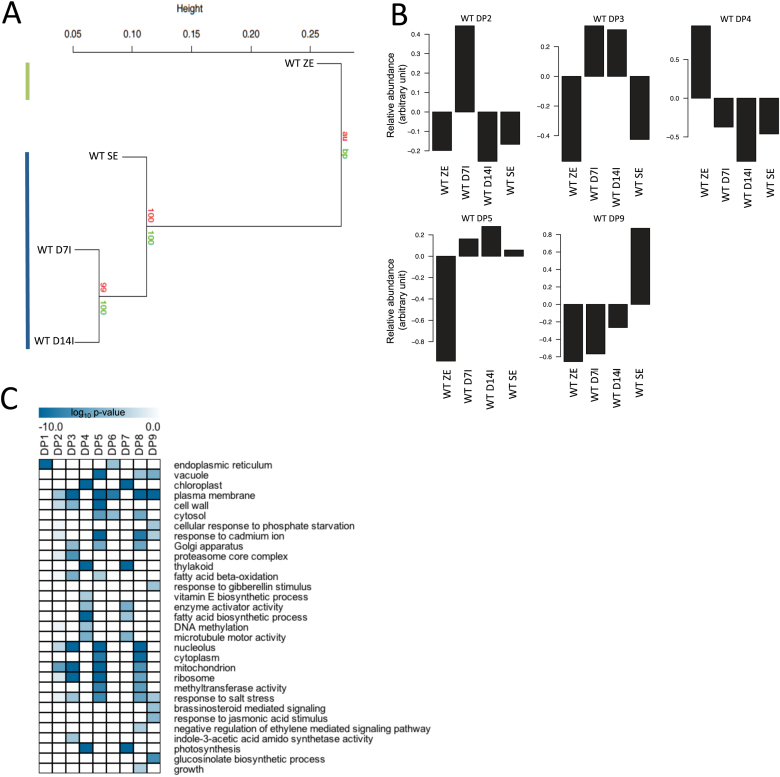
Transcriptome analysis of WT SE. (A) Global hierarchical clustering of whole GeneChip data across all stages of SE. Two distinct groups were apparent. First, for the bent-cotyledon stage of ZE (top left line) and secondly for SE (bottom left line). Bootstrap values are shown below the node and approximately unbiased values are shown above the node. (B) DPs of gene expression during WT SE. Selected clusters showed differential patterns of gene expression. Individual bars represent the relative abundance of mRNAs clustered into each DP at all stages of development. Five selected patterns are shown. (C) GO term enrichment analysis showcasing selected statistically enriched biological processes present in sets of genes from the clustering analysis. Selected GO terms are presented using a heatmap generated in TMeV (a complete list of GO terms is found in Dataset S2). GO terms are considered enriched at *p* < 0.001 where a darker colour represents a more statistically enriched term. (This figure is available in colour at *JXB* online.)

To understand global changes in gene activity during SE, we first examined the transcriptome of WT tissues during the SE process (Fig. 4). Hierarchical clustering of GeneChip data showed two distinct groups: (i) bent-cotyledon zygotic embryos, and (ii) somatic embryos (Fig. 4A). To examine how gene activity behaved in space and time, we then used our FKM clustering algorithm to identify DPs of gene expression in both space and time (Fig. 4B). Our data analysis revealed nine DPs, and the number of genes represented in each pattern varied from 476 in DP2 to 3186 in DP5 (Supplementary Dataset S2). Supplementary Fig. S4 (at *JXB* online) shows patterns where genes were expressed either in the zygotic embryo (DP4) or in a combination of stages of development during somatic embryo culture (DP2, DP3, DP5, and DP9).

To gain insight into the biological processes underlying each DP, we performed an enrichment analysis (hypergeometric distribution; *P*<0.001) of GO terms (Fig. 4C). The data showed enrichment of terms associated with photosynthesis and fatty acid biosynthesis in WT zygotic embryos, while GO terms associated with the construction of the cell were found in embryos grown in culture. For example, the terms mitochondrion, plasma membrane, and ribosome were all enriched early in the early proliferative phase of SE in DP3, while the GO terms vacuole, cell wall, nucleolus, and cytoplasm were all enriched throughout SE (DP5). Mature somatic embryos (DP9) showed enrichment for terms associated with hormone signalling and metabolism, including brassinosteroid-mediated signalling, response to jasmonic acid, and glucosinolate biosynthetic process. A complete list of genes belonging to all GO terms examined in this study can be found in Supplementary Dataset S2.

### Comparative transcriptome analysis of WT and *vtc2* tissues during SE

**Fig. 5.**
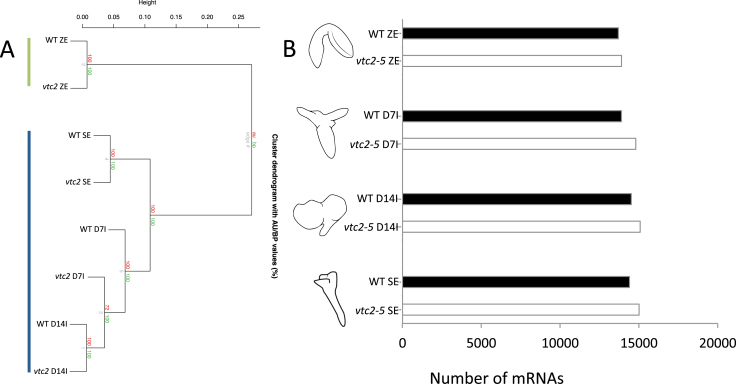
(A) Hierarchical clustering of WT and *vtc2-5* tissues during SE. Clustering analysis revealed two distinct groups: (i) bent-cotyledon zygotic embryos (top left line); and (ii) somatic embryos, including D7I, D14I and mature somatic embryos (bottom left line). (B) Number of mRNAs in WT and *vtc2-5* tissues during SE. A complete list of the mRNAs and their levels are given in Dataset S1. (This figure is available in colour at *JXB* online.)

Global mRNA populations in WT and *vtc2* tissues were compared using correlative hierarchical clustering. Regardless of genotype, similar groups were identified. Figure 5A shows the two distinct groups identified by the analysis: (i) the bent-cotyledon stage of ZE, and (ii) SE including both induction and maturation. Similar numbers of mRNAs were detected across all stages of SE using ‘Presence’ and ‘Absence’ calls according to MAS 5.0. An average of ~14 400 mRNAs (~63% of *Arabidopsis* genes on the ATH1 GeneChip) were detected at each stage of the culture process, while mRNAs tended to accumulate at a higher prevalence in *vtc2* tissue (Fig. 5B). For example, ~15 000 mRNAs were detected in *vtc2* somatic embryos compared with ~14 400 in WT mature somatic embryos (Supplementary Dataset S1).

To understand how AA deficiency affects gene activity at each stage of SE, differentially expressed genes were analysed. A total of 70 genes on the array were differentially expressed (Benjamin-Hochberg FDR<0.05, fold change >2) between WT and *vtc2* zygotic embryos. The largest number of differentially expressed genes (1281) was observed at D7I of SE where deficiencies in AA (*vtc2* tissues) repressed 648 mRNAs and induced 633 mRNAs (Supplementary Dataset S1). A large number of mRNAs (953) were also differentially expressed in mature somatic embryos (Supplementary Dataset S1).

**Fig. 6.**
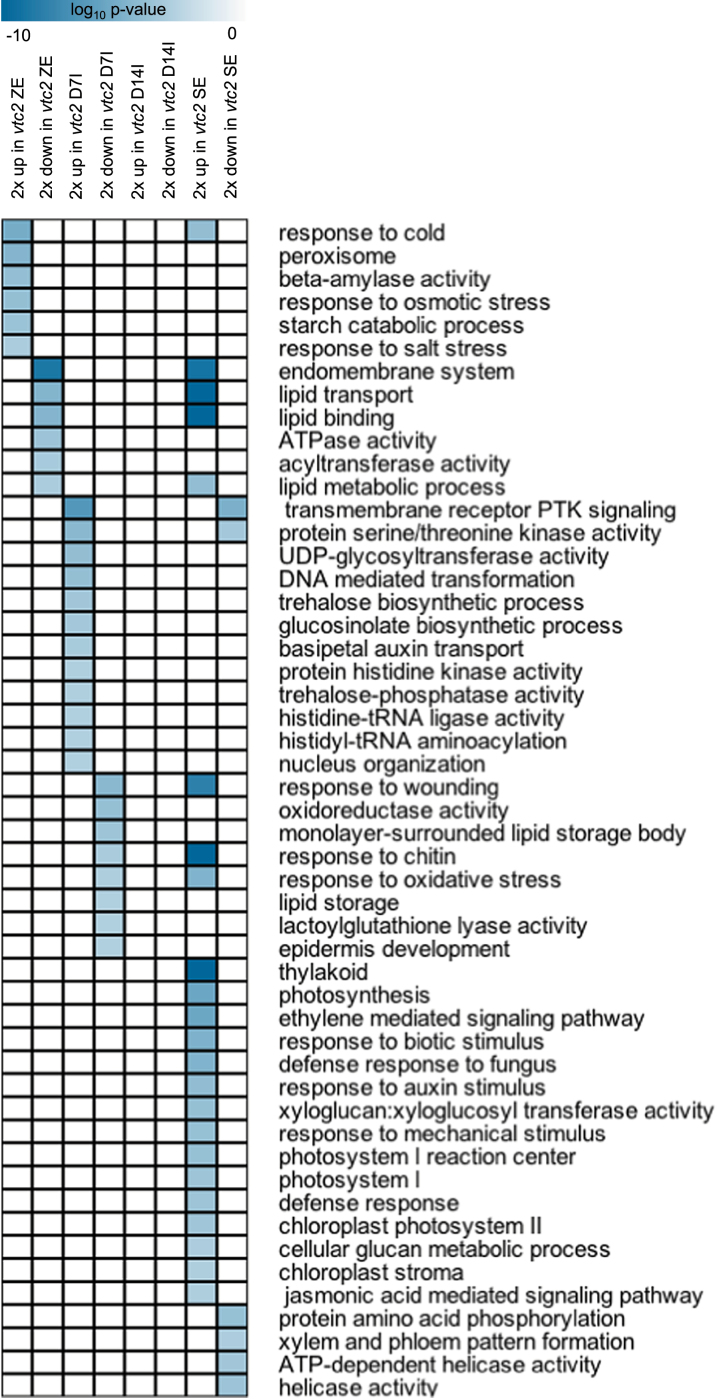
Selection of statistically enriched GO terms (*P*<0.001, hypergeometric distribution) in sets of genes that were >2-fold up- or downregulated in the *vtc2-5* mutant compared with the WT at each stage of SE. A complete list of all enriched GO terms is given in Supplementary Dataset S2. A darker colour represents greater statistical enrichment. (This figure is available in colour at *JXB* online.)

Figure 6 lists selected GO terms enriched in sets of genes either up- or downregulated in response to AA deficiency during SE (a complete list of GO terms and their enrichment values are given in Supplementary Dataset S1). At the bent-cotyledon stage of ZE, AA deficiency resulted in a selective induction of the genes in the GO terms involved in stress responses, such as peroxisome and response to osmotic stress. Included in these categories were three highly expressed cold-shock-domain proteins, *GLYCINE-RICH PROTEIN 2* (*GRP2*), *GRP7*, and *GRP8*. Starch catabolism was also enriched, whereas GO terms containing genes involved in lipid metabolic process were downregulated in *vtc2*.

**Fig. 7.**
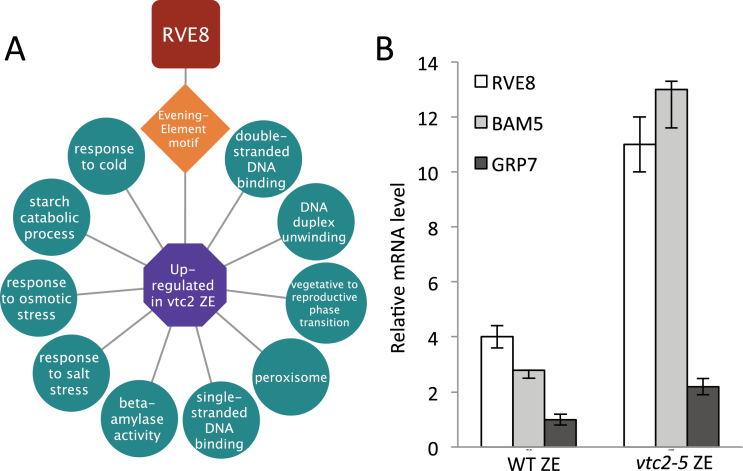
*vtc2-5* bending cotyledon zygotic embryo transcriptional module. (A) *RVE8*-Evening Element transcriptional module underlying bent-cotyledon zygotic embryos in genes upregulated in *vtc2-5* as predicted using ChipEnrich software. The mRNAs within the enriched GO terms (circles) upregulated in *vtc2-5* zygotic embryos were predicted to be under the control of the Evening Element DNA motif (diamond), which is regulated by the TF (octagon) *RVE8* (*REVEILLE8*). (B) Quantitative PCR validation of *RVE8* (*REVEILLE 8*), *BAM5* (*β-AMYLASE 5*), and *GRP7* (*GLYCINE-RICH PROTEIN 7*) gene activity found in the module. (This figure is available in colour at *JXB* online.)

To provide insight into the gene regulatory networks predicted to be operative within differentially expressed gene sets, the ‘Analysis’ tool in ChipEnrich was used to model transcriptional circuits in *Arabidopsis* (Belmonte *et al.*, 2013; Supplementary Dataset S1). This program associates lists of genes with enriched GO terms and known DNA sequence motifs with transcription factors (TFs) that are predicted or known to bind to that promoter sequence using the ATH1 GeneChip as reference. The program can then generate a module that represents such relationships. Figure 7A shows a predicted RVE8-Evening Element transcriptional module within *vtc2* zygotic embryos. This module is predicted to regulate genes associated with β-amylase activity (*β-AMYLASE 3*, At4g17090; *β-AMYLASE*, At4g15210) and genes responsive to osmotic stress and the peroxisome (*GRP7*, At2g21660; *GRP8*, At4g39260), and was validated using qRT-PCR (Fig. 7B).

At D7I, enrichment of GO terms associated with sets of genes differentially expressed in *vtc2-5* compared with WT includes basipetal auxin transport, UDP-glycosyltransferase activity, and the trehalose biosynthetic process. Several ethylene and wounding-response genes were also upregulated. In *vtc2*, other than *VITAMIN C DEFICIENT 2*, the genes presenting the greatest downregulation were *ALLENE OXIDE CYCLASE 1*/*ALLENE OXIDE CYCLASE 2*, which encode the enzymes involved in the rate-limiting step in the formation of jasmonic acid. Several genes involved in embryo development were also downregulated.

At D14I, no GO terms with a *P*<0.001 value were identified. However, numerous genes related to photosynthesis and lipid biosynthesis were upregulated in *vtc2*. Many genes that were observed to be downregulated in D7I were upregulated at D14I, including *SUCROSE SYNTHASE 2* and *FATTY ACID ELONGATION 1*. There was an abundance of differentially expressed genes with involvement in sugar signalling, with downregulation of *GALACTIONOL SYNTHASE 2*, *GLYCOSAL HYDROLASE FAMILY PROTEIN*, *THIOGLUCOHYDROLASE 1* and *UDP-GLYCOSYL TRANSFERASE 75B1*, and upregulation of *ACIDOREDUCTONE DIOXYGENASE*, *SUCROSE SYNTHASE 2*, and *TREHALOSE-6-PHOSPHATE PHOSPHATASE*.

At the maturation stage of somatic embryo development, GO terms of photosynthesis and thylakoid were statistically enriched. Genes belonging to the GO term ethylene-mediated signalling, such as *ETHYLENE FORMING ENZYME* and *ETHYLENE-RESPONSIVE ELEMENT-BINDING PROTEIN*, were also significantly increased in tissues deficient in AA, while *ETHYLENE OVERPRODUCER 1*, an inhibitor of ethylene biosynthesis, was downregulated. Genes belonging to the GO terms lipid metabolic process and lipid transport were upregulated.

### Analysis of DPs of co-expressed gene sets

**Fig. 8.**
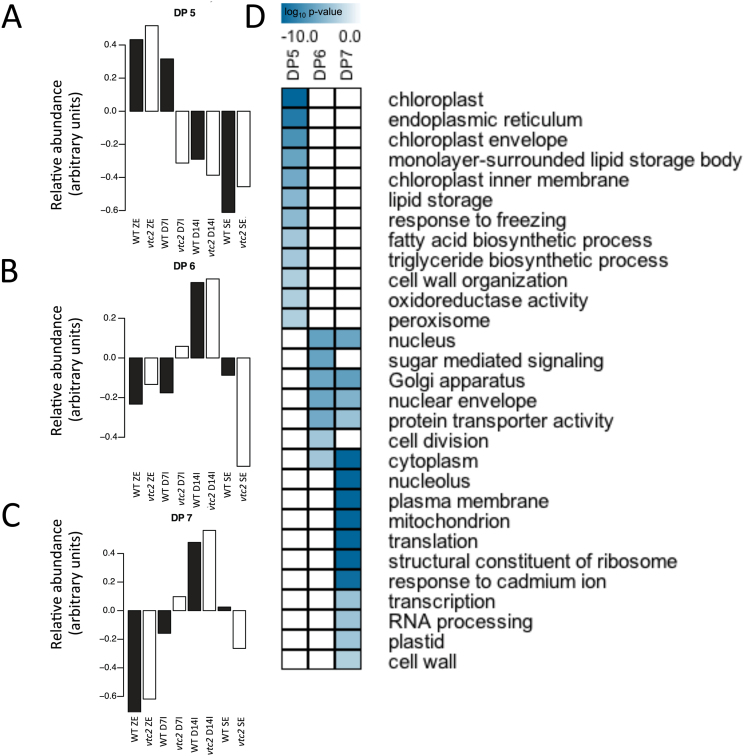
Identification of dominant patterns (DPs) of gene activity and functional categorization of genes differentially expressed between WT and *vtc2* tissues. (A) DP5 showed that gene activity in WT (filled bars) D7I was more like that of zygotic embryos compared with *vtc2-5* (open bars). (B) Accelerated gene activity in *vtc2-5* at D7I (DP6). (C) Accelerated gene activity during SE induction in *vtc2-5* (DP7). Thirteen DPs were identified using a modified FKM analysis with a Pearson correlation cut-off value of 0.85. A complete list of the mRNAs belonging to each DP is given in Supplementary Dataset S1. (D) Representative GO terms corresponding to DP5, DP6, and DP7. The listed GO terms were statistically enriched (*P*<0.001, hypergeometric distribution) between *vtc2-5* and WT tissues. A complete list of all enriched GO terms is given in Supplementary Dataset S2. A darker colour represents more statistically enriched GO terms. (This figure is available in colour at *JXB* online.)

Since the analysis presented above is limited in space to a particular stage of SE, DPs of co-expressed gene sets that vary in both space and time were identified. FKM clustering of both WT and *vtc2* datasets provided a global framework for gene activity between the two genotypes. Thirteen DPs were found following the clustering analysis with a Pearson correlation coefficient of 0.85 (Supplementary Fig. S4). The number of genes per pattern varied from 138 in DP13 to 2443 in DP8, although the majority of DPs had at least 500 genes or more (Supplementary Dataset S1). Some sets of mRNAs from *vtc2* appeared to respond more quickly to induction conditions. DP5, DP6, and DP7 showed mRNAs of D7I that were more closely related to D14I (Fig. 8), suggesting that the *vtc2* mutant had accelerated reprogramming of cellular constituents required for SE. Interestingly, a number of GO terms associated with cell signalling, proliferation, and organization were statistically enriched within these patterns, including the Golgi apparatus, nuclear envelope, and cell division. Genes belonging to the GO term sugar-mediated signalling were enriched in DP6. A large number of GO terms associated with the mitochondria were also statistically enriched during the induction phase of the SE process, suggesting that cellular respiration may be a paramount event during cell proliferation/dedifferentiation in *Arabidopsis*.

**Fig. 9.**
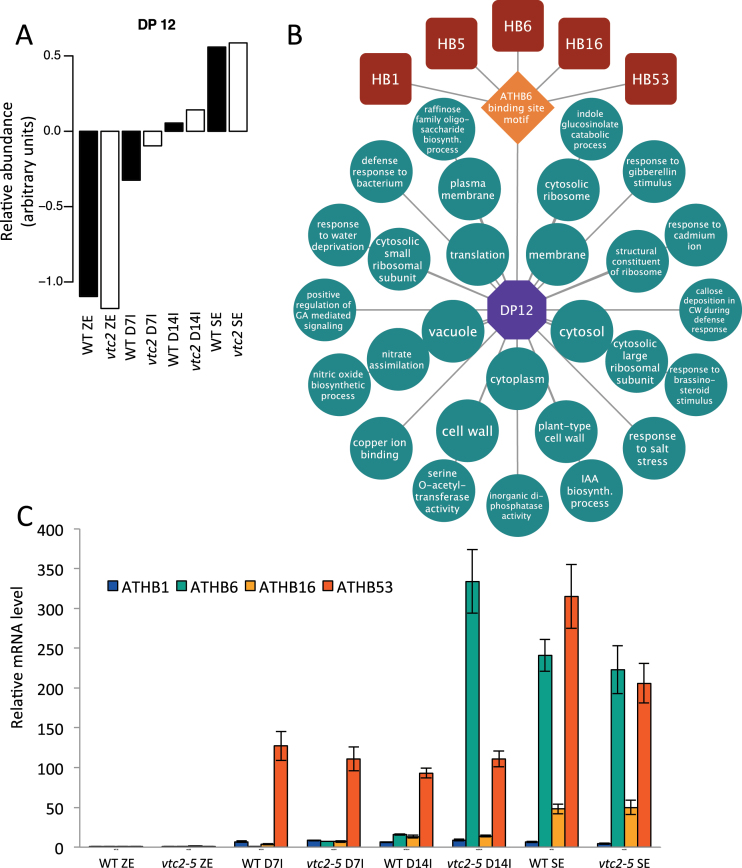
Predicted gene regulatory network controlling somatic embryo development. (A) DP12 showing the relative mRNA levels during SE in both WT and *vtc2* tissues. (B) Predicted homeobox-regulated transcriptional module underlying somatic embryo development. (C) Quantitative PCR showing increased levels of *ATHB1* (*ARABIDOPSIS THALIANA HOMEOBOX 1*), *ATHB6*, *ATHB53*, and *ATHB16* in somatic embryo tissues. (This figure is available in colour at *JXB* online.)

To gain further insight into the predicted transcriptional regulation of SE in *Arabidopsis*, a transcriptional module algorithm was used to identify possible TF regulators. DP12 showed mRNAs in *vtc2* D14I that were more similar to those of mature somatic embryos (Fig. 9A), further demonstrating an accelerated SE programme in *vtc2* mutants. The model associated *ARABIDOPSIS THALIANA HOMEOBOX* (*ATHB*) genes *ATHB1*, *ATHB5*, *ATHB6*, *ATHB16*, and *ATBH53* TFs from DP12 with the enriched ATHB6-binding site motif (Fig. 9B). This model predicted these TFs to regulate cellular components such as cell wall, vacuole, and genes that respond to ethylene and gibberellin synthesis. A complete list of enrichment data and the files required to produce this module in Cytoscape can be found in Supplementary Datasets S1–S3. The presence and relative mRNA abundance of these TFs was then validated using qPCR, and showed similar patterns of expression to the microarray (Fig. 9C).

## Discussion

The current study demonstrates how the process of SE can be improved through genetic manipulation of cellular AA levels in *Arabidopsis* using the *vtc2-5* mutant. Depletion of AA through *vtc2-5*, a previously uncharacterized allele of *VTC2*, was confirmed with qRT-PCR and enzymatic assays, and further complemented with global mRNA profiling and gene regulatory network analysis. Alterations in *vtc2-5* resulted in both an increase in callus weight and an increase in the number of somatic embryos produced per callus. The remarkable cellular and morphological characteristics of the *vtc2-5* mutant as it proceeds through SE are guided by large gene sets predicted to control a suite of biological processes in space and time.

### Effect of AA deficiency on antioxidant status during SE

A number of attempts have been made to improve the SE process through alterations to the induction or maturation medium (Ikeda‐Iwai *et al.*, 2003; George *et al.*, 2008) or through experimental changes in the cellular redox state using enzyme inhibitors (Belmonte and Stasolla, 2007). In the current study, we attempted to experimentally alter the cellular redox state using a mutant defective in vitamin C biosynthesis genes, *vtc2-5*, during SE. Over the course of SE, the *vtc2-5* mutant had a higher ASC:DHA ratio compared with WT tissues (Fig. 2B). This higher ratio was accompanied by improved callus formation, an effect that was further compounded by supplementation of the phytohormone 2,4-D (Fig. S3). While increased cellular redox ratios have been shown to promote tissue proliferation in other plant systems (Belmonte and Stasolla, 2007), to the best of our knowledge, this is the first report to show changes in the cellular ASC redox ratio during SE of *Arabidopsis*. Interestingly, enzymes responsible for maintaining a more reduced cellular environment, such as APX and DHAR, showed similar activities between WT and *vtc2* tissues (Fig. 2D–G), suggesting that the ratio between reduced and oxidized forms of AA may not be the only trigger guiding the control of cell division processes, but rather the total cellular AA levels may also be responsible. Change in ASC:DHA ratios in *vtc2-5* were probably not a result of differences in antioxidant enzyme activity (Fig. 2D–G). However, this does not preclude the possibility that changes in the AA redox state may be triggered through *de novo* biosynthesis or catabolic reactions, or through the coupling of AA to the cellular glutathione pool (Foyer and Noctor, 2011).

While the ratio of ASC:DHA was higher in *vtc2-5* tissues compared with WT, even after being transferred to maturation medium lacking 2,4-D, the large number of embryos that form per callus may be a result of an expanded *STM* mRNA domain during induction (Fig. 3D). Ectopic expression of *STM* in *Arabidopsis* has resulted in improved production of cells capable of producing somatic embryos in *Arabidopsis* and *Brassica napus* (Elhiti *et al.*, 2013), and is probably guided by a complex network of interacting partners controlling the redirection of cell fate that may involve *VTC2*.

### SE is associated with large shifts in gene activity in WT and *vtc2-5* tissues

Global mRNA profiling of SE in WT tissues showed major shifts in gene activity during the culturing process and may be a result of (i) auxin treatment to induce callus formation; and (ii) auxin removal to induce somatic embryo maturation (Fig. 4A). Changes in gene activity were further scrutinized using enrichment analysis and a number of GO terms and genes associated with cell development and organization were found to be enriched early in the induction phase, supporting the structural changes noted above. When comparing WT with *vtc2-5* deficient tissues, hierarchical clustering analyses showed that the genotypes tended to cluster based on developmental stage (Fig. 5A), and within the induction group, it was clear that, at the genetic level, tissues deficient in *VTC2* levels were more similar to the maturation phase of the WT somatic embryos. While similar numbers of genes were expressed at all stages of SE regardless of genotype, it was still not clear what sets of genes were responsible for the rapid induction of cell proliferation or the improved number of somatic embryos formed per callus.

### Bent-cotyledon zygotic embryos

Some of the differences observed early in the SE process may be explained by differential expression of gene activity in the *vtc2-5* mutant at the zygotic embryo stage compared with WT zygotic embryos. AA-deficient zygotic embryos appeared to be under a general stress response through genes associated with the cold, osmotic, and salt stress response (Table 1, Fig. 6). Gliwicka *et al.* (2013) found that the SE process was associated with the expression of many stress-response TFs in *Arabidopsis*, probably due to the placement of the zygotic embryo in culture, since SE induction is believed to be a stress response to *in vitro* culturing conditions. The upregulation of stress-related genes in the *vtc2* mutant may allow these zygotic embryos to become embryogenic competent sooner than WT zygotic embryos during induction.

**Table 1.** Selection of differentially expressed genes in vtc2-5 tissues compared with WT levels across somatic embryogenesis in Arabidopsis thaliana

Zygotic embryos				Day 14 induction (cont.)	
Gene	Name	Fold change	Gene	Name	Fold change
**AT4G38680**	GRP2 (Cold-shock domain protein)	12.75	AT2G26400	ARD (Acireductone dioxygenase)	3.07
**AT2G21660**	ATGRP7 (Cold, circadian rhythm)	4.90	AT5G51470	Auxin-responsive GH3 family protein	3.00
**AT4G39260**	ATGRP8 (Cold, circadian rhythm)	4.20	AT4G34520	FAE1 (Fatty acid elongation)	2.74
**AT2G35840**	sucrose-phosphatase 1 (SPP1)	2.73	AT3G56400	WRKY70 (WRKY DNA-binding protein 70)	2.65
**AT4G17090**	BAM8 (β-amylase 8)	2.63	AT4G08780	Peroxidase superfamily protein	2.37
**AT4G15210**	BAM5 (β-amylase 5)	2.04	AT5G10100	Trehalose-6-phosphate phosphatase, putative	2.31
**AT1G19640**	JMT (JA carboxyl methyltransferase)	–2.68	AT5G49190	SUS2 (Sucrose synthase 2)	2.13
**AT4G30960**	CIPK6 (CBL-interacting protein kinase)	2.56	AT5G66700	ATHB53 (Homeobox-53)	2.06
**AT5G42180**	PER64 (Peroxidase 64)	–4.75	AT3G22490	LEA (Late embryogenesis abundant protein)	–2.02
**AT2G48130**	LTP (Lipid transfer protein)	–4.88	AT1G56600	GOLS2 (Galactinol synthase 2)	–2.36
**AT1G19900**	Glyoxal oxidase-related	–7.00	AT5G17220	GSTF12 (Glutathione S-transferase)	–2.40
**AT4G26850**	VTC2 (Vitamin C defective 2)	–11.71	AT2G44460	Glycosyl hydrolase family 1 protein	–2.91
**AT2G23540**	GDSL-motif lipase/hydrolase family protein	–12.76	AT3G60140	SEN2 (Dark inducible 2)	–3.38
**AT4G38080**	Hydroxyproline-rich glycoprotein family	–16.12	AT4G26850	VTC2 (Vitamin C defective 2)	–26.06
**Day 7 induction**			**Mature somatic embryos**		
**Gene**	**Name**	**Fold change**	**Gene**	**Name**	**Fold change**
**AT5G63660**	LCR74 (Low-weight cysteine-rich 74)	8.89	AT5G38170	LTP (lipid transfer protein) family protein	59.02
**AT4G09940**	AIG1 (Avirulence induced gene)	6.29	AT1G31580	ECS1 (Glutamate-cysteine ligase)	26.96
**AT3G43190**	SUS4 (Sucrose synthase 4)	5.89	AT2G34870	MEE26 (Maternal effect embryo arrest 26)	11.80
**AT4G08770**	Peroxidase, putative	5.72	AT5G27200	ACP5 (Acyl carrier protein 5)	10.99
**AT3G23000**	CIPK7 (CBL-interacting protein kinase 7)	5.46	AT1G22590	MADS-box family protein	10.60
**AT3G05890**	RCI2B (Rare-cold inducible 2B)	4.40	AT5G59320	LTP3 (Lipid transfer protein 3)	9.87
**AT4G33560**	ETR2 (Ethylene response 2)	4.33	AT4G35770	SEN1 (Dark inducible 1)	8.62
**AT1G70290**	TPS8 (Trehalose phosphatase /synthase 8)	3.98	AT1G74670	Gibberellin-responsive protein, putative	8.12
**AT5G23020**	2-Isopropylmalate synthase	3.98	AT3G62550	Universal stress protein (USP) family protein	7.08
**AT5G58750**	Wound-responsive protein-related	3.62	AT5G26000	TGG1 (Thioglucoside dehydrolase 1)	6.87
**AT1G52150**	ATHB15 (Homeobox-15)	2.70	ATCG00020	Photosystem II reaction centre core component	6.70
**AT5G66700**	ATHB53 (Homeobox-53)	2.58	AT4G17490	ERF6 (Ethylene-responsive element binding 6)	5.73
**AT2G17870**	Cold-shock DNA-binding family protein	2.51	ATCG01060	PsaC subunit of photosystem I.	5.26
**AT5G20830**	SUS1 (Sucrose synthase 1)	2.48	AT2G38470	WRKY33 (WRKY DNA-binding protein 33)	5.07
**AT2G22420**	ERS2 (Ethylene response sensor 2)	2.47	AT5G18600	Glutaredoxin family protein	4.89
**AT1G20440**	COR47 (Cold regulated 47)	2.33	AT4G23810	WRKY53 (WRKY DNA-binding protein 53)	4.83
**AT2G22420**	PER17 (Peroxidase 17)	2.25	AT5G01870	Lipid transfer protein, putative	4.77
**AT5G65310**	ATHB5 (Homeobox-5)	2.22	AT3G56400	WRKY70 (WRKY DNA-binding protein 70)	4.74
**AT5G10100**	Trehalose-6-phosphate phosphatase, putative	2.06	AT2G46400	WRKY46 (WRKY DNA-binding protein 46)	4.46
**AT2G38470**	WRKY33 (WRKY DNA-binding protein 33)	–2.14	AT5G49190	SUS2 (Sucrose synthase 2)	4.43
**AT5G10140**	FLC (Flowering locus C)	–2.35	ATCG00140	ATPase subunit	4.10
**AT3G23250**	MYB15 (Myb domain protein 15)	–2.83	AT3G51600	LTP5 (Lipid transfer protein 5)	4.08
**AT5G49190**	SUS2 (Sucrose synthase 2)	–3.40	**AT3G25760; 70**	AOC1; AOC2 (Allene oxide cyclase 1 and 2)	4.08
**AT4G34520**	FAE1 (Fatty acid elongation)	–3.68	AT1G06080	ADS1 (Delta 9 desaturase 1)	3.57
**AT3G26790**	FUS3	–3.99	AT5G47230	ERF5 (Ethylene-responsive element binding 5)	3.42
**AT5G45830**	DOG1 (Delay of germination 1)	–4.56	AT5G23940	EMB3009 (Embryo defective 3009)	3.30
**AT3G22490**	LEA (Late embryogenesis abundant protein)	–4.95	AT1G05010	EFE (Ethylene forming enzyme)	3.29
**AT5G57550**	XTR3 (Xyloglucan endotransglycosylase 3)	–5.88	AT3G23250	MYB15 (Myb domain protein 15)	3.11
**AT3G25760; 70**	AOC1; AOC2 (Allene oxide cyclase 1 and 2)	–8.72	ATCG00160	Chloroplast ribosomal protein S2	2.95
**AT4G26850**	VTC2 (Vitamin C defective 2)	–37.97	AT1G19610	LCR78 (Low-weight cysteine-rich 78)	2.81
	**Day 14 induction**		AT4G15210	BAM5 (β-amylase 5)	2.50
**Gene**	**Name**	**Fold change**	AT1G53885	Senescence-associated protein-related	
**AT5G38170**	LTP (lipid transfer protein) family protein	6.57	AT4G17090	BAM8 (β-amylase 8)	2.34
**ATCG00680**	CP47(Subunit of photosystem II)	5.64	AT1G21970	LEC1 (Leafy cotyledon 1)	2.07
**ATCG00430**	Subunit K of NADH dehydrogenase	4.70	AT1G52150	ATHB15 (Homeobox-15)	–2.03
**ATCG01060**	PsaC subunit of photosystem I.	4.18	AT3G51770	ETO1 (Ethylene overproducer 1)	–2.09
**ATCG00140**	ATPase subunit	4.01	AT2G44460	Glycosyl hydrolase family 1 protein	–2.18
**ATCG00490**	Large subunit of RUBISCO.	3.69	AT1G80410	EMB2753 (Embryo defective 2753)	–2.41
**AT2G34870**	MEE26 (Maternal effect embryo arrest 26)	3.59	AT3G22490	LEA (Late embryogenesis abundant protein)	–2.66
**AT1G06080**	ADS1 (Delta 9 desaturase 1)	3.39	AT4G26850	VTC2 (Vitamin C defective 2)	–35.42
**ATCG00330**	30S Chloroplast ribosomal protein	3.37			

The transcriptional module algorithm was used to predict the transcriptional circuitry controlling different sets of genes between *vtc2-5* and WT tissues. The strategy to associate TFs with enriched DNA sequence motifs within sets of genes that control various biological processes provides an integrated framework for the identification of molecular mechanisms controlling plant development using large-scale data sets (Belmonte *et al.* 2013). Thus, the functional differentiation between the two genotypes may lie with the TFs controlling different sets of genes underlying the different stages of SE. The data revealed a predicted RVE8-Evening Element transcriptional module controlling sets of genes responsible for the stress response, in addition to genes encoding enzymes responsible for the breakdown of starch (Fig. 7A). The *vtc2* mutant exhibited greater *RVE8* expression compared with the WT, suggesting that AA deficiency, through either alterations in redox state or interactions through other pathways, is affecting the expression of this TF and, consequently, its targets. Lai *et al.* (2012) demonstrated that crosstalk between redox and circadian rhythm pathways are commonplace. It was determined that genes influencing redox state, including those for the production of AA, are under the regulation of clock genes, and that expression of clock genes are affected by the redox state. The presence of the genes within the *RVE8* module were validated using qRT-PCR, but functional analyses of these genes in SE should provide the necessary information required to better understand the transcriptional circuits underlying the earliest stages of this process.

### Induction

While many methods of inducing SE have been reported, not all the embryo-like structures develop fully or properly (Ikeda-Iwai *et al.*, 2003). Low yields are a common problem in SE, often resulting in extensive manipulation to the induction medium, such as supplementation with plant hormones or other stress factors (Gaj, 2004; Potters *et al.*, 2007). This study is unique in that internal stress is applied without the requirement for external stimuli. This would be an economic and long-term method to improve SE production in the absence of tedious modifications of the chemical environment of the culture. We believe that the majority of the differences in phenotype observed in the *vtc2-5* mutant are the result of the impact of AA deficiency on the induction phase of SE.

Indeed, the induction phase appears to be when the most differences in cell anatomy and gene activity between WT and *vtc2* somatic embryos occur. This suggests AA deficiency, in the presence of auxin, affects cellular metabolism and signalling, leading to enhanced callus production. These results are consistent with studies showing that auxin is necessary to initiate SE by supporting cell proliferation (Wójcikowska *et al.*, 2013), and that, combined with the reduced cellular redox status of AA, provides optimal cues for callus induction.

Our data shows a coordinated cell signalling programme is operative at the level of the GeneChip in actively dividing cells (Fig. 6). Along with increased callus size (Fig. 1B) and embryo production (Fig. 1C), differences in ultrastructure in *vtc2* cells were also observed in the induction phase. The appearance of endomembranous activity (Fig. 3G) suggests the cells are re-entering the cell cycle and actively dividing. Sheahan *et al.* (2007) reported that characteristic features of plant cells that are about to divide include the redistribution of the endoplasmic reticulum, and an increase in the number or volume of Golgi. The authors identify the function of extensive Golgi bodies as participating in the formation of new cell plate material in dividing cells. This is consistent with our data that showed increased cell division patterns in *vtc2* tissues (Fig. 3B) compared with their WT counterparts (Fig. 3A). Indeed, numerous vesicles could be found throughout the cytoplasm of *vtc2* cells (Fig. 3G).

The ability to accelerate cell division processes has important implications in plant development. Gene sets associated with accelerated cell division and developmental programmes (DP6 and DP7) showed upregulation of sugar-mediated signalling pathways and the enrichment of mitochondra GO terms in *vtc2-5* tissues (Fig. 7) and suggested that nutrients are being mobilized and energy is being produced to support the rapid cell division that occurs during induction. This is supported by anatomical evidence showing an increased prevalence of mitochondria (Fig. 3I) and the reduction in chloroplast starch content (Fig. 3H) in the *vtc2* mutant compared with WT (Fig 3E, F).

*vtc2-5* tissues may also be inherently more responsive to auxin. A relationship between auxin utilization and cellular redox states has been demonstrated in multiple systems (Hirt, 2000; Bashandy *et al.*, 2010; Iglesias *et al.*, 2010). It is possible that, due to the decreased pool of AA, *vtc2* cells are more sensitive to changes in redox state via the action of auxin, or that perhaps the reduced ASC:DHA ratio predisposes the cells to greater proliferation rates and is acted upon synergistically via auxin signalling. Auxin transport in the basipetal direction may provide the conditions necessary to promote cell division in younger tissues (Friml and Palme, 2002; Lewis *et al.*, 2007). Alternatively, *vtc2-5* tissues may exhibit an increased production of endogenous auxin. The relationship between auxin and the control of cellular redox switches controlling development warrants further investigation.

### Maturation

The maturation phase of SE represents a major shift in gene activity and appears to be accelerated in *vtc2-5*. Similarly to the AA-deficient zygotic embryo, the mature *vtc2-5* SE appears to have an elevated stress response when compared with WT. GO terms including response to cold, response to wounding, response to biotic stimulus, and defence response were all enriched in *vtc2-5* (Fig. 6). This suggests that AA deficiency continues to impart a stress in maturing embryos, and may be involved in the increased number and quality of somatic embryos being formed.

*LEC1*, a known regulator of embryo maturation (Yang and Zhang, 2010), was shown to be upregulated during SE maturation (Table 1), and is consistent with the results of Gruszczyńska and Rakoczy-Trojanowska (2011) and Ledwoń and Gaj (2011), who showed that *LEC1* activity is associated with embryogenic culture in rye and *Arabidopsis*. Since the accumulation of oil bodies coincides with embryo maturation, the upregulation of *LEC1* due to lower AA levels may be responsible for the production of the abundant oil bodies observed in *vtc2-5* somatic embryos (Fig. 3G) and is in agreement with the results of Mu *et al.* (2008) who showed that overexpression of *LEC1* in *Arabidopsis* increases fatty acid biosynthesis.

Several genes belonging to the enriched GO terms ethylene-mediated signalling pathway, jasmonic acid-mediated signalling pathway, and response to auxin were also abundant in *vtc2-5*, suggesting that a complex interaction between cellular redox state and hormone crosstalk is operative during tissue differentiation. While mutations in *vtc2* led to improved SE in *Arabidopsis* (Fig. S1), it is still not clear whether other mutations in the AA biosynthetic pathway may also exhibit similar phenotypes. If this is the case, it can be speculated that AA deficiency may improve SE in general and that increased cellular levels of AA may prevent ectopic signalling processes inhibitory to cell fate or cell survival.

### Gene regulatory networks underlying SE in *Arabidopsis*

The prediction and identification of transcriptional regulators guiding SE provide an opportunity to further dissect the molecular mechanism controlling development. The analysis presented here revealed a number of homeobox TFs predicted to control SE differentiation through regulatory pathways not found in zygotic embryos. Homeobox genes have long been associated with plant development (Hake *et al.*, 2004), and have recently been exploited in plants to improve SE in a number of species including *Arabidopsis* (Mordhorst *et al.*, 2002), canola (Elhiti *et al*., 2010, 2013), soybean (Ma *et al.*, 1994), cotton (Bouchabké-Coussa *et al.*, 2013), and spruce (Belmonte *et al.*, 2007). However, the targets of these TFs have largely remained unsolved. Here, a model is presented in which a set of homeodomain leucine zipper TFs (ATHB1, ATHB5, ATHB6, ATHB16, and ATHB53) regulates sets of genes responsible for structural components of the embryo including the cell wall, plasma membrane, and vacuole, and may also control genes that respond to phytohormones such as gibberellic acid or brassinosteroids, and through signalling molecules such as nitric oxide – all through the ATHB6-binding site motif (a complete list of predicted gene targets is found in Supplementary Dataset S2). While validation of the microarray data with qRT-PCR of the homeobox TFs confirmed their relative abundance within somatic embryos (Fig. 9C), this model is strictly predictive and requires further exploration through loss- or gain-of-function mutants and chromatin immunoprecipitation experiments.

### Conclusions

This study provides evidence that endogenous levels of AA affect the regulation of growth and development during SE. It is possible that the cellular pool of AA may serve as a signal to mediate changes in gene expression through the coordination of gene regulatory networks. Large-scale changes in gene activity were observed between and across genotypes; however, AA deficiency through alterations in *VTC2* levels appears to promote an exaggerated auxin response, especially during callus formation, and improved the number of embryos produced per explant. Finally, the integrated transcriptional module analysis provides the framework for the identification and discovery of new transcriptional regulators of SE in *Arabidopsis*.

## Supplementary data

Supplementary data may be found at JXB online.

Supplementary Table S1. List of primer sequences used to validate T-DNA insertions and qRT-PCR experiments.

Supplementary Dataset S1. Correlation table of GeneChips, MAS 5.0 data, fold change gene lists, DP gene lists and primers used for qRT-PCR experiments.

Supplementary Dataset S2. List of GO terms, DNA sequence motifs, and their enrichment.

Supplementary Dataset S3. Network and attributes files required for network visualization software Cytoscape.

Supplementary Fig. S1. (A) Gene structure of At4g26850 (*VTC2*) and insertion location for SALK_146824 and SALK_076245. (B) qRT-PCR validation of low *VTC2* mRNA levels in leaf and silique tissues of SALK_146824 (*vtc2-5*) and SALK_076245 (*vtc2-6*). (C) Percent of zygotic embryos forming callus. (D) Percentage of calli forming somatic embryos. (E) Number of mature SEs formed per explant.

Supplementary Fig. S2. (A, B) Effect of ascorbate (ASC) on somatic embryo development in WT and *vtc2-5*. ASC was supplemented in the induction medium; 10, 100, and 1000 µM ASC rescued the number of zygotic embryos capable of producing callus (A) and the frequency of explants forming somatic embryos (B). (C) Number of somatic embryos per explant.

Supplementary Fig. S3. (A) Effect of 2,4-D (0–4.5 μM) on somatic embryo induction. (B) The frequency of explants that form somatic embryos. (C) The number of mature somatic embryos formed per explant.

Supplementary Fig. S4. Dominant patterns of gene expression during SE in WT (filled bars) and *vtc2-5* (open bars).

Supplementary Data

## Abbreviations:

2,4-D: 2,4-dichlorophenoxyacetic acid
AA: ascorbic acid
APX: ascorbate peroxidase
ASC: ascorbate
ATHB: *Arabidopsis thaliana* homeobox
D7I: 7 d on induction medium
D14I: 14 d on induction medium
DHA: dehydroascorbate
DHAR: dehydroascorbate reductase
DP: dominant pattern
FDR: false discovery rate
FKM: fuzzy K means
FW: fresh weight
GO: Gene Ontology
GR: glutathione reductase
LEC: *LEAFY COTYLEDON*
MDHAR: monodehydroascorbate reductase
SAM: shoot apical meristem
SD: standard deviation
qRT-PCR: quantitative reverse transcription-PCR
RMA: Robust Multichip Average
SE: somatic embryogenesis
STM: SHOOTMERISTEMLESS
TEM: transmission electron microscopy
TF: transcription factor
VTC: *VITAMIN C DEFICIENT*
WT: wild-type
ZE: zygotic embryogenesis.
