# Supplementary material for: Vitamin C deficiency improves somatic embryo development through distinct gene regulatory networks in Arabidopsis
Source: J Exp Bot. 2014 Aug 23;65(20):5903–18. doi: 10.1093/jxb/eru330 (PMC4203126; doi:10.1093/jxb/eru330)
Supplement: Supplementary Data [file supp_65_20_5903__index.html]

Vitamin C deficiency improves somatic embryo development through distinct gene regulatory networks in Arabidopsis — Supplementary Data 

# Vitamin C deficiency improves somatic embryo development through distinct gene regulatory networks in *Arabidopsis*

## Supplementary Data

Data files

**Files in this Data Supplement:**

- Supplementary Data - Supplementary Data
- Supplementary Data - Supplementary Data
- Supplementary Data - Supplementary Data
- Supplementary Data - Supplementary Data
