# Supplementary material for: Vitamin C deficiency improves somatic embryo development through distinct gene regulatory networks in Arabidopsis
Source: J Exp Bot. 2014 Aug 23;65(20):5903–18. doi: 10.1093/jxb/eru330 (PMC4203126; doi:10.1093/jxb/eru330)
Supplement: Supplementary Data [file supp_eru330_jexbot127084_file004.pdf]

Vitamin C deficiency improves somatic embryo development through distinct gene regulatory networks in *Arabidopsis*

Michael F Becker, Ainsley Chan, Xingyu Mao, Ian J Girard, Samantha Lee, Mohamed Elhiti, Claudio Stasolla, and Mark F Belmonte

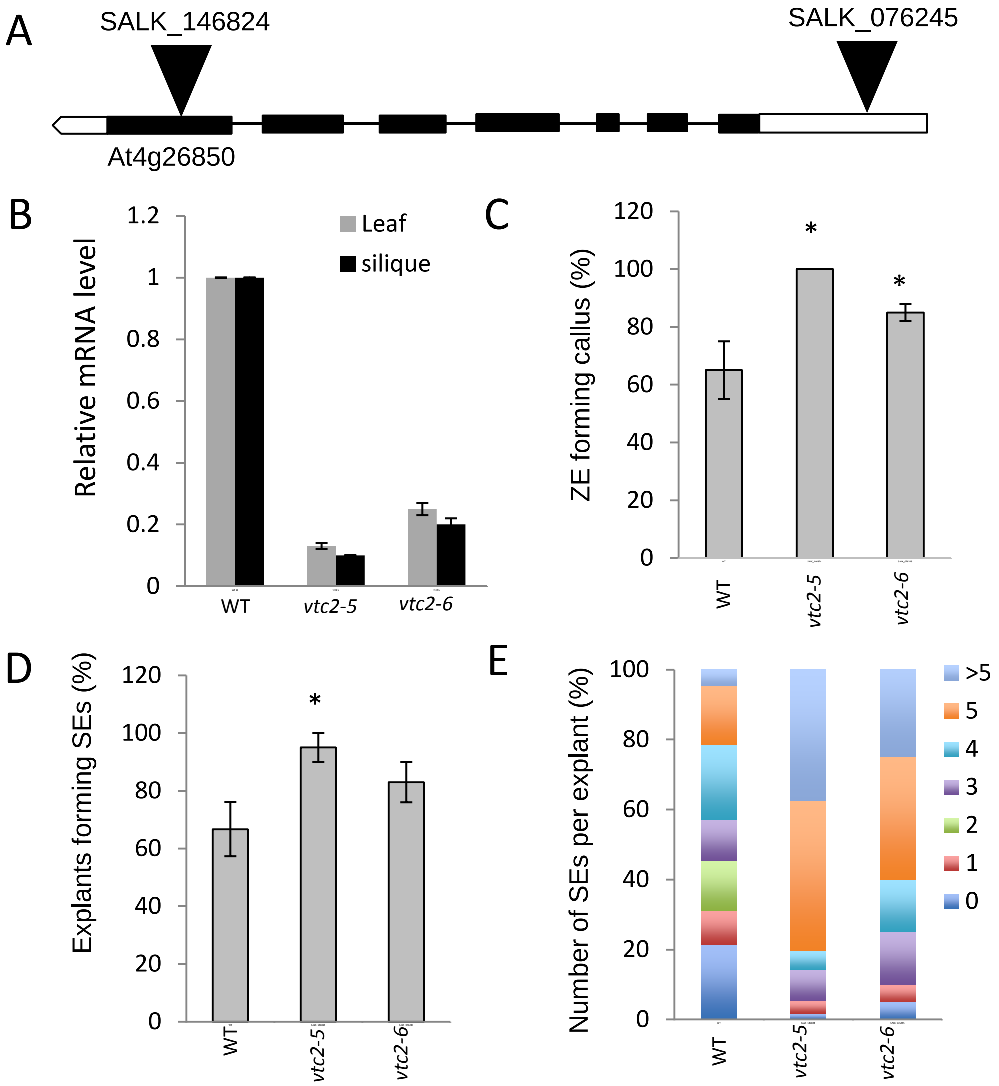

Fig. S1. A. Gene structure of At4g26850 (*VTC2*) and insertion location for SALK_146824 and SALK_076245. B. qRT-PCR validation of low *VTC2* mRNA levels in leaf and silique tissues of SALK_146824 (*vtc2-5*) and SALK_076245 (*vtc2-6*). C. Percent of zygotic embryos forming callus. D. Percentage of calli forming somatic embryos, and E. Number of mature SEs formed per explant. Asterisks above each bar indicate values that are significantly different ($P<0.05$) from WT value. SE, somatic embryo; ZE, zygotic embryo.

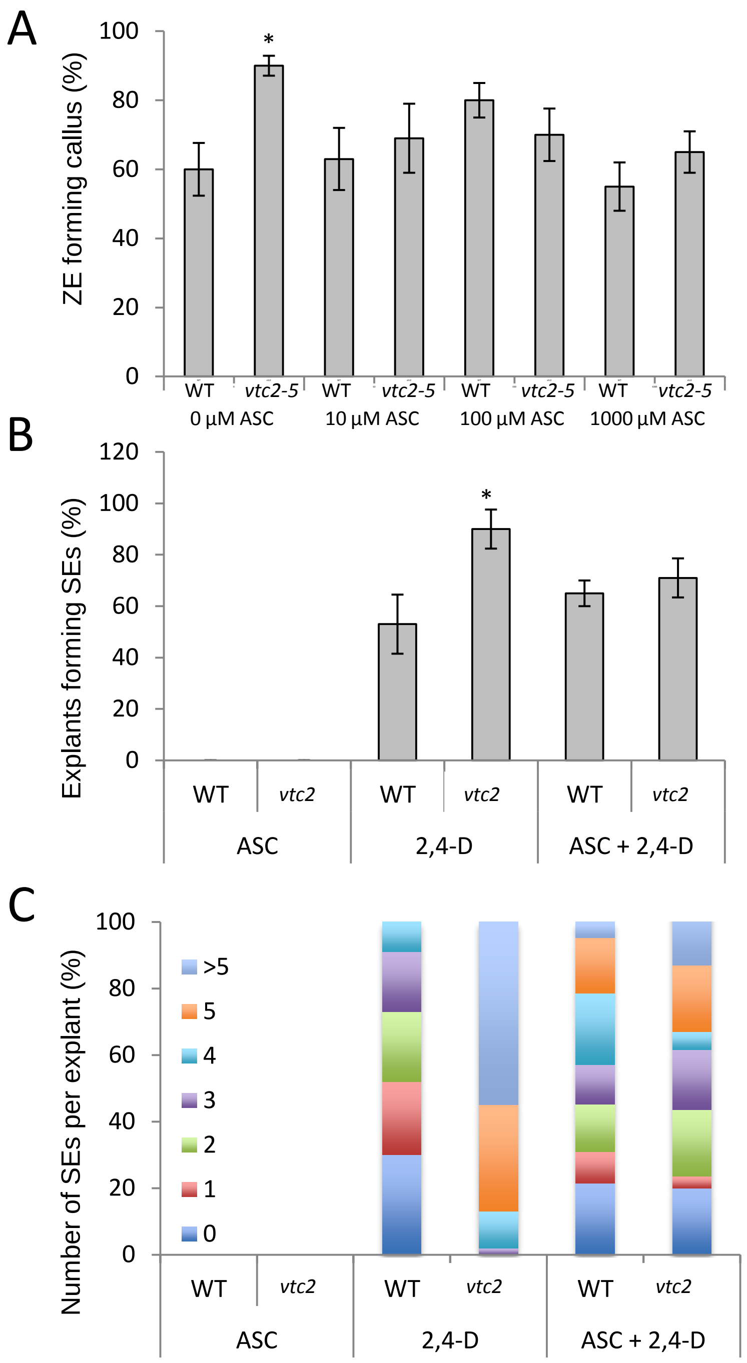

Fig. S2. Effect of ascorbate (ASC) on SE development in WT and *vtc2-5*. ASC was supplemented in the induction media. 10, 100, 1000 µM ASC rescued the number of ZEs capable of producing callus (A) and the frequency of explants forming SEs (B). (C) Number of SEs per explant. Asterisks above each bar indicate significant difference from control values (P<0.05) ± SD. Abbreviations are as in materials and methods.

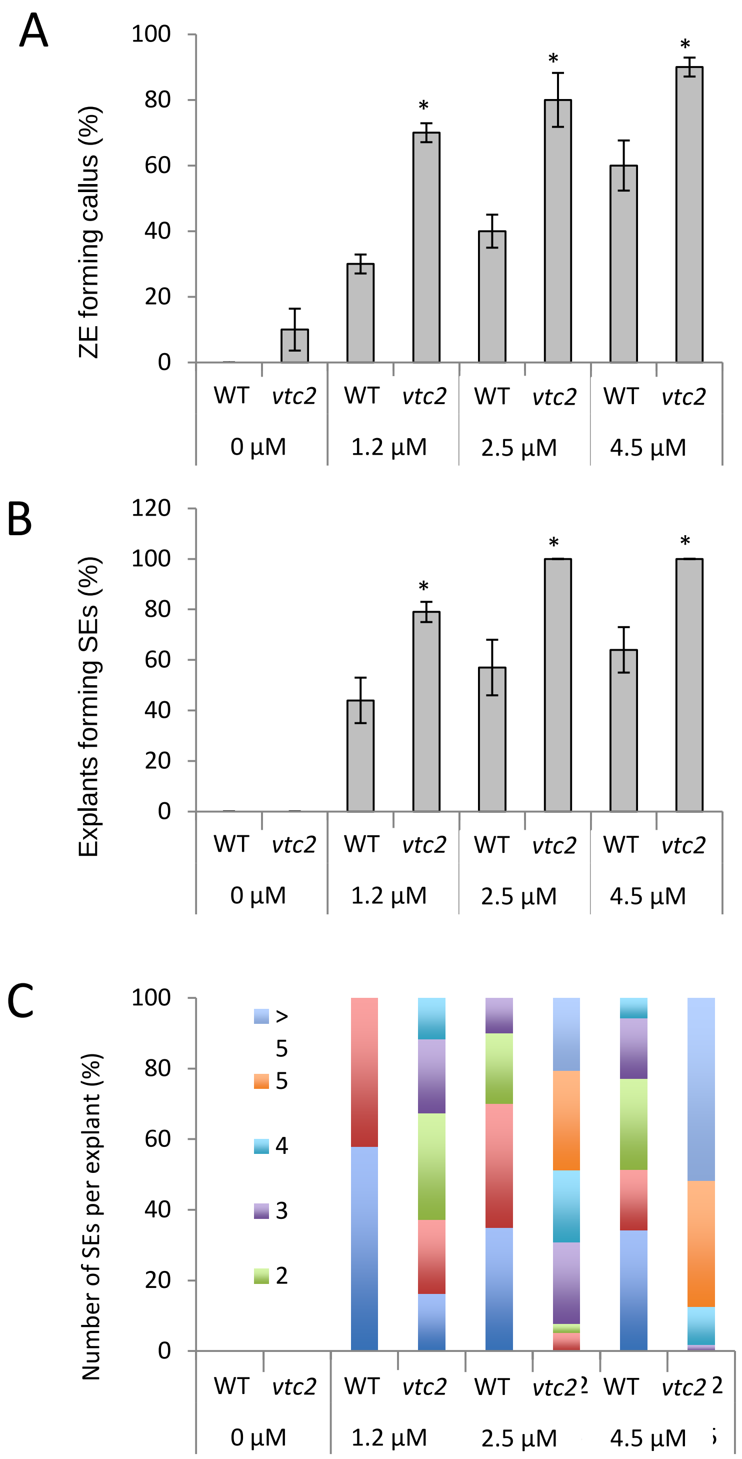

Fig. S3. A. Effect of 2,4-D (0-4.5 µM) on SE induction. B. The frequency of explants that form SEs. C. The number of mature SEs formed per explant. ± SD, asterisks above each bar indicate significant difference from control values (P<0.05). Abbreviations are as in materials and methods.

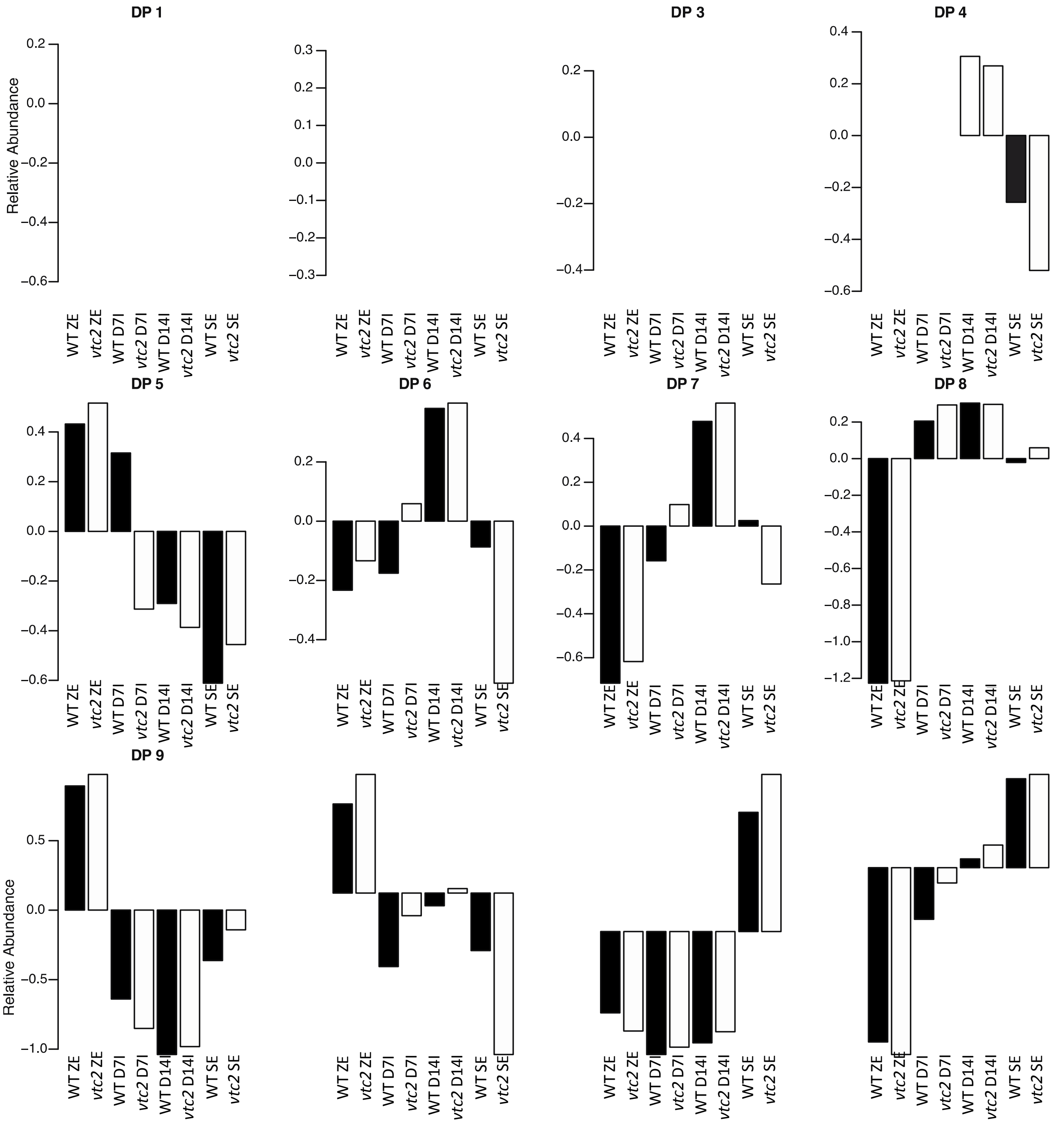

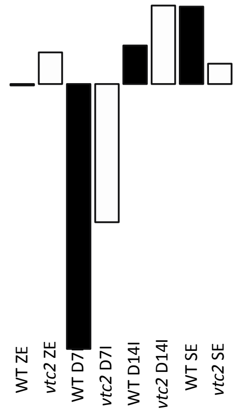

Fig. S4. Dominant patterns of gene expression during SE in WT (black bars) and *vtc2-5* (white bars). Thirteen DPs were identified using a modified fuzzy K means analysis with a Pearson correlation cutoff of 0.85. A complete list of mRNAs belonging to each DP is found in Dataset S2. Bars represent the relative mRNA abundance found in each sample and is an arbitrary unit.
